# Tracing SARS-CoV-2 clusters across local scales using genomic data

**DOI:** 10.1073/pnas.2501435122

**Published:** 2025-08-07

**Authors:** Leke Lyu, Mandev Gill, Guppy Stott, Sachin Subedi, Cody Dailey, Gabriella Veytsel, Magdy Alabady, Kayo Fujimoto, Ryker Penn, Pamela Brown, Roger Sealy, Justin Bahl

**Affiliations:** ^a^Institute of Bioinformatics, University of Georgia, Athens, GA 30602; ^b^Center for the Ecology of Infectious Diseases, University of Georgia, Athens, GA 30602; ^c^Department of Infectious Diseases, College of Veterinary Medicine, University of Georgia, Athens, GA 30602; ^d^Department of Epidemiology and Biostatistics, College of Public Health, University of Georgia, Athens, GA 30602; ^e^Department of Statistics, University of Georgia, Athens, GA 30602; ^f^Department of Plant Biology, University of Georgia, Athens, GA 30602; ^g^Department of Health Promotion and Behavioral Sciences, The University of Texas Health Science Center at Houston, Houston, TX 77030; ^h^Houston Health Department, Houston, TX 77054

**Keywords:** viral evolution, genomic epidemiology, pandemic control

## Abstract

A quantitative understanding of local transmission dynamics is essential for designing effective prevention strategies. In this study, we developed a computational workflow to identify viral introductions and trace locally circulating clusters. We analyzed over 26,000 SARS-CoV-2 genomes and their associated metadata, collected between January and October 2021, to explore introduction and local dispersal patterns in Greater Houston, a major metropolitan area known for its demographic diversity. Our analysis identified more than 1,000 independent introduction events, resulting in clusters of varying sizes. The majority of introductions originated from domestic sources, while international introductions occurred earlier and were associated with larger cluster sizes. An analysis of locally circulating clusters revealed age-structured transmission dynamics. Geographic reconstruction of cluster spread identified Harris County as the primary viral source for surrounding areas. The outbreak in the source population was characterized by 1) a smaller proportion of new cases associated with external viral imports and 2) longer persistence times of circulating lineages. Overall, our high-resolution spatiotemporal reconstruction of the epidemic provides essential insights into the local-scale transmission landscape, supporting outbreak-specific, regional response strategies and public health planning.

## Significance

Pathogen genome sequencing has become a critical tool for outbreak response, leading to a rapid increase in the amount of available sequence data. Here, we present an analytical workflow to trace imported SARS-CoV-2 clusters through communities using large-scale genome datasets. Our approach pinpoints when, where, and how many introductions occurred, while also tracking the circulation of resulting clusters. By incorporating metrics such as the Source Sink Score, Local Import Score, and Persistence Time, our analysis reveals transmission heterogeneity between subregions of the focal area. These insights are essential for monitoring viral introductions, guiding targeted control measures, and enhancing the ability of local responders to address the challenges of current epidemics and future pandemics.

Genomic epidemiology has greatly enhanced our understanding of emerging infectious diseases and informed efforts to control their spread (1–3). In the context of SARS-CoV-2, previous research has demonstrated its capability to clarify the virus’s origins and spread (4, 5), reconstruct local transmission chains (6), assess the effectiveness of nonpharmaceutical interventions (7), and identify key predictors of viral lineage movements (8). These epidemiological insights, translated from the virus’s evolutionary history, are crucial for shaping public health policies and would not have been possible without extensive sequencing efforts. By August 2024, over 16 million genome sequences had been submitted to the Global Initiative on Sharing All Influenza Data (GISAID) (9). Although these expansive COVID-19 datasets facilitate high-resolution inferences about local transmission dynamics, they also present significant computational challenges (10). In response, new algorithms, software, and computational workflows have emerged since the onset of the pandemic, including tools for rapid phylogenetic tree construction (11, 12) and the Thorney BEAST module for more efficient generation of time-resolved trees (13, 14).

Houston, the largest city in the Southern United States, anchors the Greater Houston metropolitan area and is one of the most demographically diverse cities in the country (15). It is also one of the most economically segregated cities, marked by sharp divisions in income, education, and occupation (16). According to Covid Act Now, Houston faces considerable challenges due to its high population density, significant proportion of non-English speakers, and notable income disparity. These factors strain the city’s health system, making Houston more vulnerable to a SARS-CoV-2 outbreak than over 90% of US metropolitan areas (17). This vulnerability highlights the urgent need to understand local patterns of SARS-CoV-2 dispersal and how these patterns vary across different socioeconomic regions.

The COVID-19 pandemic has been characterized by the emergence and spread of genetically distinct virus variants that exhibit increased transmissibility compared to earlier lineages (18). The Delta variant, in particular, not only spreads more rapidly (19) but also leads to higher rates of hospitalization (20) and demonstrates greater immune evasion (21) than the previously dominant Alpha variant. In Houston, the emergence of Delta occurred in a context of heterogeneous prior immunity, resulting from both previous infections and vaccinations. The first case of the Delta variant in Houston Methodist Hospital was identified in mid-April 2021, during a period of declining COVID-19 cases (22). Beginning in early July, there was a sharp increase in cases driven by the Delta variant, with these cases doubling in frequency approximately every 7 d (22). Several critical questions remain unanswered, which cannot be resolved without genomic epidemiological inference. What is the primary source of these variants? What role did Houston play in the introduction of Delta to the United States?

With the support of the Houston Health Department (HHD), we accessed an extensive dataset comprising over 10,000 Delta genomes sampled from Houston between January 2021 and October 2021, each linked with metadata such as zip code, age, and sex. This dataset offers a valuable opportunity to investigate transmission dynamics in Houston, examine how population structure influences disease spread, and assess variations in SARS-CoV-2 transmission across different subregions.

A notable phylodynamic workflow developed by Dellicour et al. (23) facilitates large-scale phylogeographic analysis through two principal steps: First, a preliminary discrete trait analysis (24) on fixed empirical topologies identifies introduction events; second, it estimates the circulation dynamics of local viral clusters (25, 26). We adapted this analytical workflow to examine the spatial invasion dynamics of SARS-CoV-2 in Greater Houston, as illustrated in *SI Appendix*, Fig. S1. Applying a unique algorithm, we determined the timing and number of viral introductions and tracked the resulting circulating clusters. We specifically investigated whether international or domestic introductions played a more prominent role. Additionally, we modeled the transmission structure among different demographic groups, determined by sex and age. Finally, we explored the spatiotemporal variation in viral dispersal across various subregions, defined here as counties, within Greater Houston.

## Results

### Identifying Distinct Introduction Events andLocally Circulating Clusters.

**Fig. 1.**
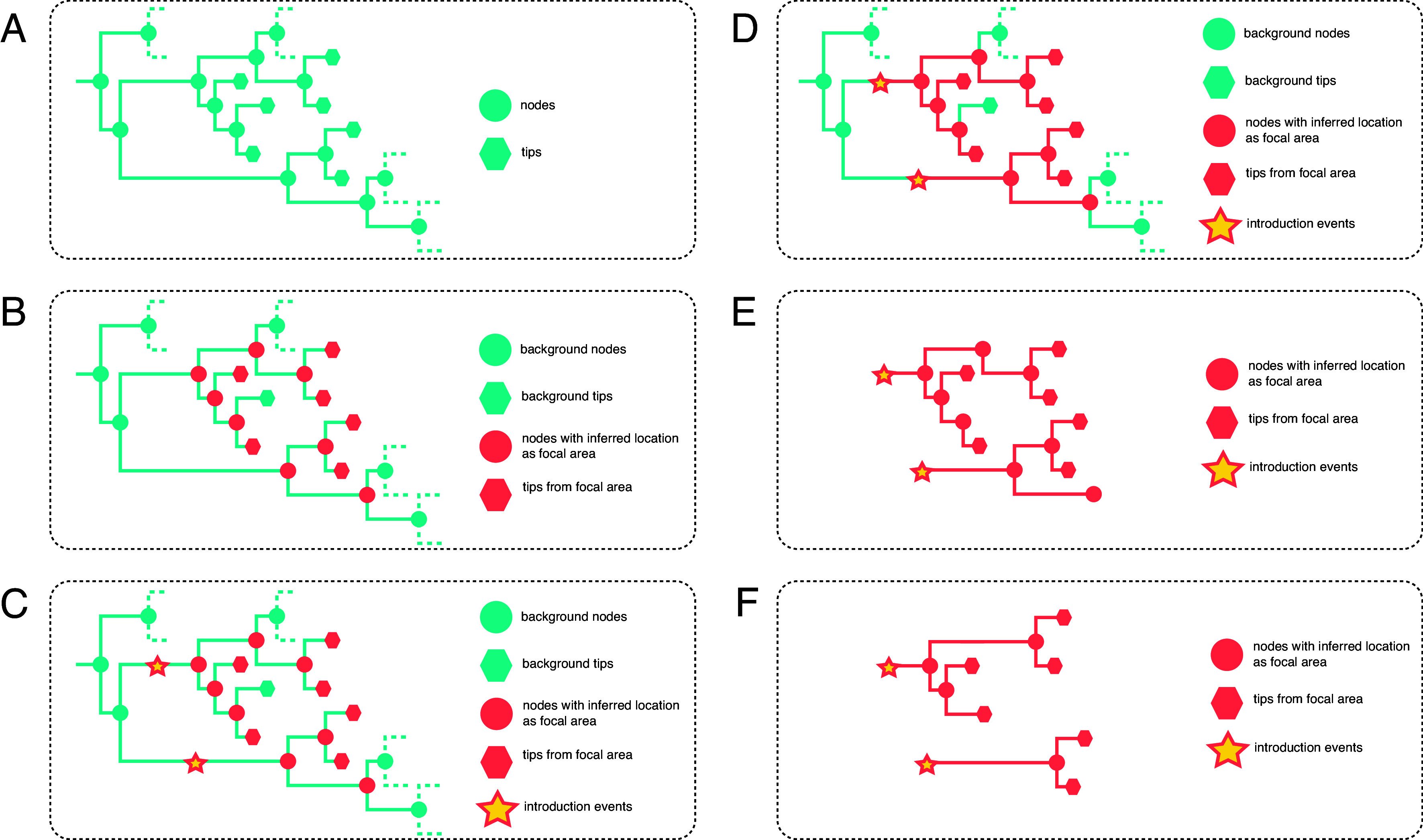
Outline of the methodology for extracting locally circulating clusters. (*A*) Phylogeny depicting isolates sampled from the focal area and background regions. (*B*) Discrete trait analysis inferring geographic trait states for internal nodes. (*C*) Identification of introduction events on branches connecting background nodes to nodes/tips from the focal area. (*D*) Clade extension from introduction nodes to encompass all nodes and tips from the focal area. (*E*) Overview of pseudoclusters before refinement. (*F*) Final locally circulating clusters obtained by collapsing branches connected to single-descent nodes.

Our dataset comprised 26,138 complete SARS-CoV-2 genomes, including 9,186 sampled from Houston and 16,952 contextual sequences from around the world. We performed a discrete phylogeographic reconstruction on a time-calibrated phylogeny, assigning internal nodes to one of three locations: “Houston,” “Domestic,” or “International.” Introduction events were defined as transitions along branches where the inferred location changed from “non-Houston’ (i.e., Domestic or International) to Houston. Subtrees descending from these introduction events were extracted and pruned to represent locally circulating clusters (Fig. 1).

**Fig. 2.**
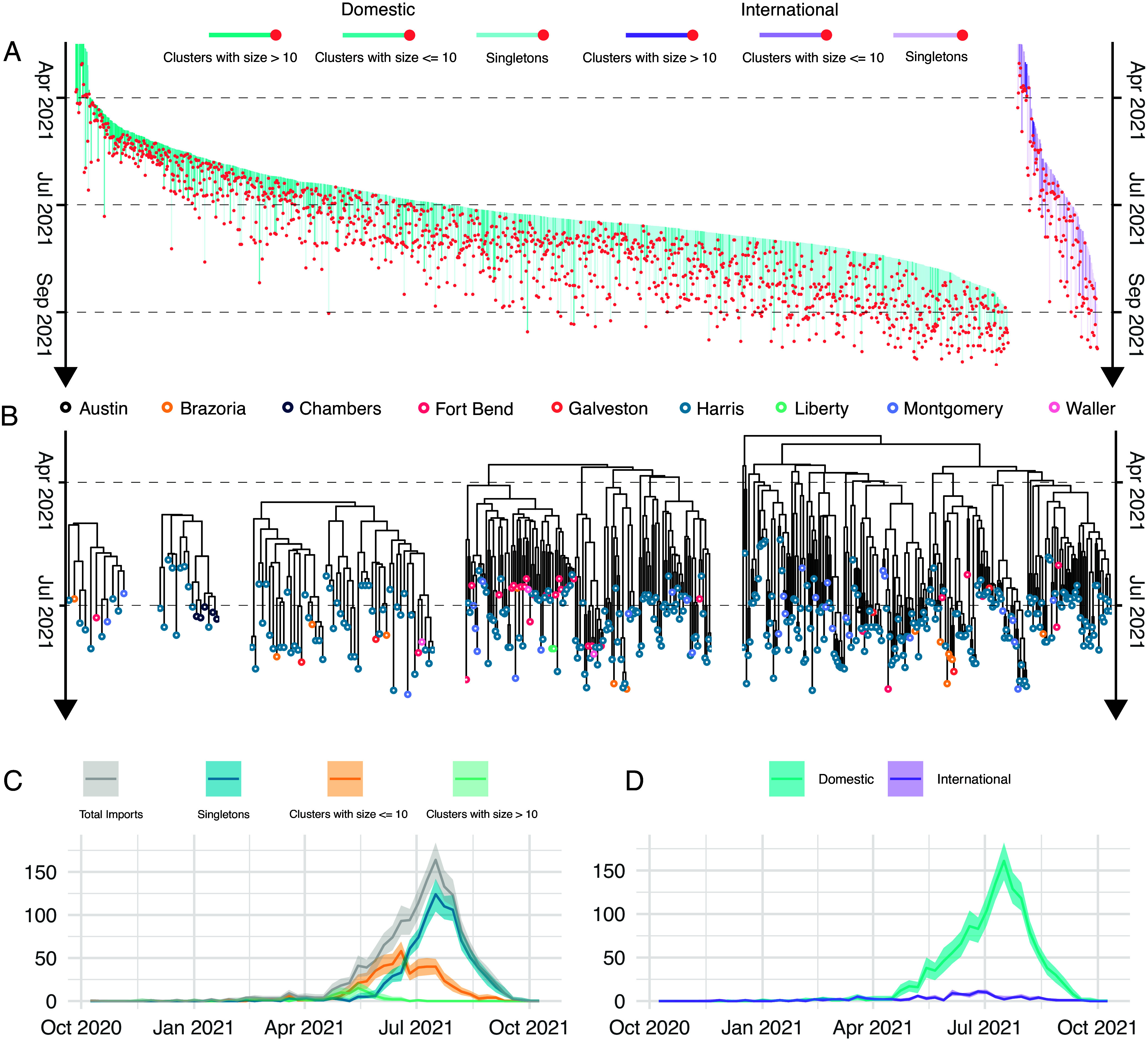
Dynamics of viral introduction into Greater Houston. (*A*) 1,479 SARS-CoV-2 introduction events into Houston inferred from a time-scaled phylogeny. Each vertical line represents an inferred introduction of SARS-CoV-2 into Houston from either domestic (blue) or international (purple) sources. These lines correspond to introduction branches in the phylogeny. These are branches that connect non-Houston nodes to Houston nodes or tips. The introduction time is assumed to be the midpoint of each branch. The *Top* of each line marks this estimated introduction time, while the *Bottom* connects to the Houston node or tip. Lines are semitransparent, with greater opacity indicating larger downstream transmission clusters. (*B*) Local dispersal patterns in Houston are illustrated using five randomly selected clusters. Tips are colored by the county where isolates were sampled. (*C*) Frequency of viral introductions per week, with curves colored by the size of resulting local transmission lineages. Shaded regions represent the 95% HPD intervals. (*D*) Frequency of viral introductions per week, with curves colored to distinguish between domestic and international sources. Shaded regions represent the 95% HPD intervals.

We identified 1,479 independent introduction events [95% highest posterior density (HPD): 1,402 to 1,556]. The sizes of the resulting clusters were skewed, with most introductions (909 events; 95% HPD: 853 to 968) resulting in singletons (Fig. 2*A*). For further analysis of local transmission dynamics, we selected 82 clusters, each comprising more than 10 sequences. Together, these clusters accounted for 6,455 sequences from the Greater Houston area. The two largest clusters contained 2,159 and 1,878 sequences. To illustrate patterns of local dispersal, we randomly selected five representative clusters, shown in Fig. 2*B*. Using a deep learning approach (27), we inferred transmission parameters—basic reproduction number ($R_{0}$) and infectious period—directly from time-scaled phylogenies. Notably, the two largest clusters did not exhibit higher $R_{0}$ values than smaller clusters, suggesting that cluster persistence and size were not determined by increased transmissibility or fitness (*SI Appendix*, Fig. S2 and Table S1).

Temporal trends showed that earlier introductions were more likely to give rise to larger clusters (Fig. 2*C*). For example, during EPI Week 17, 55.6% of introductions formed clusters larger than 10 cases, 33.3% formed clusters with fewer than 10 cases, and 11.1% were singletons. By EPI Week 30, this distribution had shifted where 86.2% of introductions resulted in singletons, 13.8% in small clusters, and none in clusters larger than 10.

We classified viral imports into Houston as either domestic or international. Early in the outbreak, introductions were scattered from both sources. However, after late April, domestic imports increased and became the dominant type, while international imports peaked earlier (Fig. 2*D*). In total, we identified 1,359 domestic imports (95% HPD: 1,279 to 1,432), significantly outnumbering 119 international imports (95% HPD: 109 to 132). We modeled the observed cluster size distribution using a negative binomial distribution, which accounts for overdispersion in transmission (28). This allowed us to estimate the mean cluster size (r) and the dispersion parameter (k), where lower k indicates greater variability in cluster sizes and a stronger role for superspreading. Clusters arising from international imports were, on average, larger (r = 22.33) and more variable in size (k = 0.27) (Table 1).

**Table 1.** Characterizing cluster size distributions with the dispersion parameter (k) and mean cluster size (r)

Parameter	All Sources (Median, 95% HPD)	Domestic Sources (Median, 95% HPD)	International Sources (Median, 95% HPD)
k	0.48 (0.46 to 0.50)	0.60 (0.50 to 0.98)	0.27 (0.23 to 0.40)
r	6.21 (5.90 to 6.55)	4.77 (3.22 to 5.91)	22.33 (9.25 to 40.35)

Smaller k values imply a high degree of variability, often driven by superspreading events.

### Phylogeny-Trait Correlation Among Locally Circulating Clusters.

**Fig. 3.**
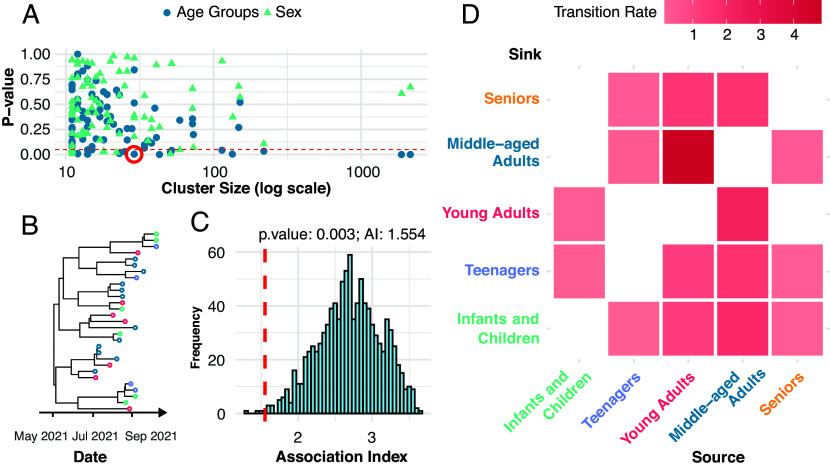
Demographic determinants of transmission. (*A*) Association between 82 locally circulating clusters and demographic traits. Blue circles represent statistical test results for age groups, and green triangles represent results for sex groups. Shapes below the red line (*P* < 0.05) indicate clusters significantly associated with the corresponding demographic trait. (*B*) Detail view of a highlighted cluster (circled in panel *A*), with tip colors representing age groups. (*C*) Null distribution of the association index for the highlighted cluster, with the observed value indicated by a red dashed line. (*D*) Discrete trait diffusion model results for age groups, shown as a heatmap. Only transitions with Bayes Factor (BF) > 30 are displayed. Cell colors represent transition rates, with darker shades indicating higher rates. The same color scheme denoting age groups was used in panels *B* (tip colors) and *D* (heatmap).

We investigated the correlations between phylogenetic structures and demographic traits, specifically age and sex, to better understand how these factors influence transmission dynamics (Fig. 3*A*). Age groups were categorized as follows: infants and children (0 to 12 y), teenagers (13 to 18 y), young adults (19 to 35 y), middle-aged adults (36 to 55 y), and seniors (56 y and older). These age classifications were adapted from widely used demographic frameworks in public health and sociology.

For each locally circulating cluster (Fig. 3*B*), we quantified phylogeny-trait correlations using the Association Index (29, 30). To evaluate the significance of these correlations, we generated null distributions of the Association Index by randomizing trait assignments across phylogeny tips 1,000 times (Fig. 3*C*). This approach allowed us to perform the tip-trait association test. The null hypothesis assumed that traits were randomly associated with phylogenetic structures. A low *P*-value (*P* < 0.05) rejected this hypothesis, indicating a strong association and suggesting limited viral dispersal between different trait groups.

We tested 82 locally circulating clusters for associations with demographic traits (Fig. 3*A*). Among these, 20 clusters exhibited significant correlations between age group traits and phylogeny (*P* < 0.05). In contrast, only 6 clusters showed significant correlations for sex traits. Overall, sex groups appeared more interspersed on the phylogeny, indicating that viral transmission is more constrained within age groups than within sex groups.

### Demographic Determinants of Transmission.

Assuming 1) each local transmission cluster resulted from a single introduction into Houston, 2) all sequences within a cluster represented local transmission, and 3) each cluster was an independent observation of the same population process, we jointly estimated a single discrete trait model across all 82 circulating clusters to quantify viral dispersal among age groups. The analysis included 6,455 isolates distributed across the following age groups: 2,009 young adults, 1,995 middle-aged adults, 841 infants and children, 818 seniors, 771 teenagers, and 21 individuals of unknown age.

Our model (Fig. 3*D*) identified young and middle-aged adults as the primary drivers of viral transmission. These groups were the sources of the highest transition rates, including: from young adults to middle-aged adults (4.813 transitions per year) and vice versa (2.216 transitions per year); from middle-aged adults to infants and children (1.895 transitions per year), seniors (1.518 transitions per year), and teenagers (1.516 transitions per year); and from young adults to seniors (1.459 transitions per year), teenagers (1.216 transitions per year), and infants and children (1.202 transitions per year). A complete list of diffusion rates between age groups is provided in *SI Appendix*, Table S2. Notably, the main sources of transmission to the vulnerable group—seniors—were teenagers, young adults, and middle-aged adults (BF > 100).

### Heterogeneous Dynamics of Viral Dispersal in Subregions of Greater Houston.

**Fig. 4.**
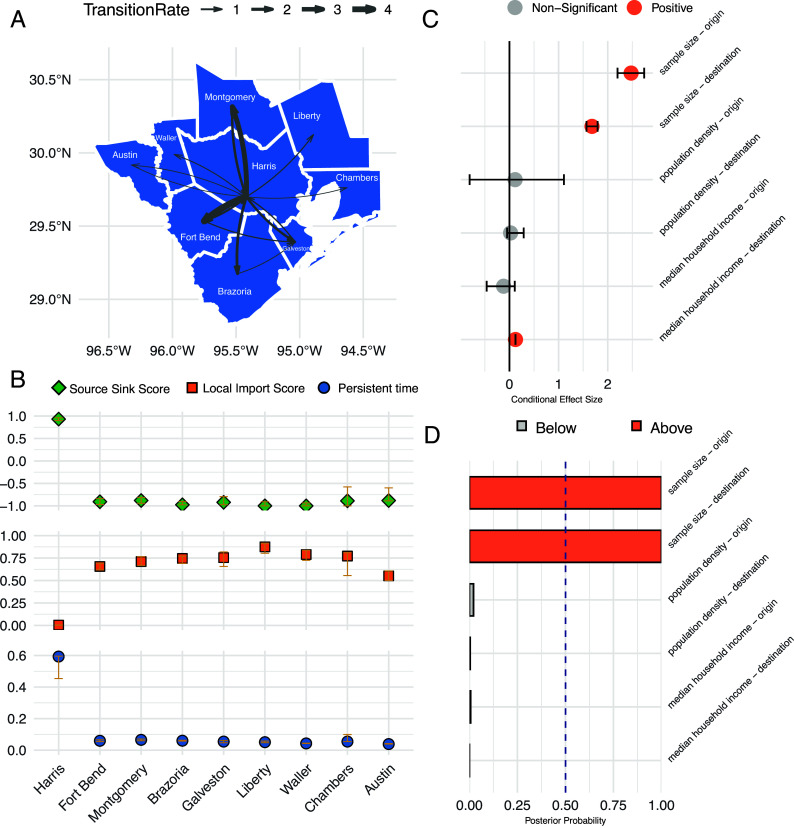
Distinct transmission patterns in subregions of Greater Houston. (*A*) Discrete phylogeographic reconstruction of the dispersal history across 9 counties. Arrow thickness indicates the magnitude of transition rates. All transitions shown on the map were decisively supported by Bayes factors (>100) (*B*) Source Sink Scores, Local Import Scores, and Persistence Time across these subareas. In these bar charts, golden error bars represent the associated 95% HPD, providing a measure of uncertainty for each score. Source Sink Score ranges from 1 (viral source) to −1 (viral sink). Local Import Score ranges from 0 (epidemic is locally maintained) to 1 (epidemic relies on introduction). (*C*) Conditional effect size of covariates within the generalized linear model (GLM). Conditional effect size represents the effect size of the variable coefficient given inclusion in the GLM. The black line represents a conditional effect size of 0, signifying little impact of the covariate on viral dispersal. (*D*) Inclusion probabilities of covariates in the GLM, with a threshold set at a posterior probability of 0.5.

We estimated a jointly fitted discrete model to reconstruct the geographic dispersal history of SARS-CoV-2 in Greater Houston. Categorizing location traits into 9 counties, the joint model (Fig. 4*A*) identified 14 transitions decisively supported by Bayes Factors (>100). The highest transition rate was from Harris County to Fort Bend County (4.569 transitions per year), followed by transitions from Harris County to Montgomery County (3.292) and Harris County to Brazoria County (1.642). A detailed list of rates is provided in *SI Appendix*, Table S3.

Discrete trait analysis yielded a sample of trees from the posterior distribution with resolved geographic states of internal nodes, enabling the calculation of metrics that summarize the transmission pattern of each subregion. These metrics include 1) Source Sink Score (31), which identifies populations as either viral sources or sinks based on net viral flow weighted by outbreak size, 2) Local Import Score (31), which assesses the relative influence of viral introductions versus local transmission in driving the epidemic, and 3) Persistence Time (32–34), which is the average duration (in years) that a lineage remains within its sampled location, traced backward through the phylogeny. Harris County (Fig. 4*B*) was identified as a key source of viral dispersal in Houston (Source Sink Score: 0.931, 95% HPD: 0.899 to 0.961). The epidemic in Harris County was predominantly driven by local transmission rather than viral introductions (Local Import Score: 0.004, 95% HPD: 0.002 to 0.006). Viral lineages also demonstrated longer persistence in Harris County (Persistence Time: 0.593 y, 95% HPD: 0.454 to 0.596 y). In contrast, viral sinks exhibited distinct transmission patterns, characterized by higher Local Import Score and shorter Persistence Time. To test the robustness of these findings, we conducted two additional joint estimates: one using only the two largest clusters (*SI Appendix*, Fig. S3 and Table S3) and another using the remaining 80 smaller clusters (*SI Appendix*, Fig. S4 and Table S3). Both analyses supported Harris County as the primary viral source, characterized by the lowest Local Import Score and the longest Persistence Time.

We extended the discrete trait diffusion model by incorporating a GLM framework to assess the association between demographic variables and transition rates between counties. The following predictors were considered: sample count, case count, population size, population density, median household income, and accumulated death count (*SI Appendix*, Table S4). Principal component analysis (PCA) revealed that sample count, case count, population size, and death count clustered together, indicating collinearity among these variables (*SI Appendix*, Fig. S5). Among them, we retained sample count as the representative predictor, as it showed the weakest correlation with the two other variables of interest—median household income and population density (*SI Appendix*, Fig. S6). The following predictors were statistically supported (Fig. 4 *C* and *D*): 1) sample count of the origin county (Conditional Effect Size: 2.476; 95% HPD: 2.200 to 2.742; Inclusion Probability: 1) and 2) sample count of the destination county (Conditional Effect Size: 1.680; 95% HPD: 1.568 to 1.794; Inclusion Probability: 1). We interpret the sample-count effect as a proxy for outbreak magnitude: Counties experiencing larger epidemics both exported and imported more viral lineages.

## Discussion

The global spread of SARS-CoV-2 has triggered new outbreaks, but most cases arise from local transmission. Understanding local transmission dynamics quantitatively is essential for designing effective prevention strategies. In this study, we developed a computational workflow to identify locally circulating clusters from phylogenetic data, incorporating contextual sequences. This approach allows us to assign each isolate from the focal area to a specific cluster, enabling a detailed analysis of the timing and frequency of introductions. The resulting clusters are essential for inferring local transmission patterns.

In collaboration with the HHD, we analyzed over 26,000 SARS-CoV-2 genomes and their associated metadata to study how the virus was introduced and spread within Greater Houston. This major metropolitan area is known for its demographic diversity. Our analysis covered the period between January and October 2021 and identified 1,479 independent introduction events (95% HPD: 1,402 to 1,556). Domestic origins were the predominant source of these introductions overall (Fig. 2). We also examined how demographic structures influenced the spread of the virus. The tip-trait association test suggested that viral transmission was more restricted between age groups than between sexes (Fig. 3*A*). Furthermore, discrete trait analysis modeled transmission between different age groups (Fig. 3*D*). Finally, we reconstructed the spatiotemporal spread of locally circulating clusters (Fig. 4). Our analysis revealed that transmission dynamics varied across subregions of Greater Houston. For example, in Harris County—a key viral source—introductions accounted for a smaller proportion of new cases, while transmission chains within the county tended to last longer than those in other counties.

Our analysis quantitatively confirms that the Delta outbreak in Houston was driven by multiple independent introduction events. These introductions seeded extensive local transmission, resulting in clusters of varying sizes. Notably, earlier introductions tended to give rise to larger clusters. This pattern is consistent with previous findings on COVID-19 introductions in the United Kingdom (8, 35), New York City (36), and Denmark (5). At the start of the outbreak, we observed that scattered introductions came from both domestic and international sources. However, after late April, domestic introductions increased sharply and became the dominant driver of new cases. This shift suggests that Houston was not a primary entry point for SARS-CoV-2 into the United States, unlike states such as New York, California, and Florida, which were identified as major entry hubs (4). Another molecular epidemiological study of the Delta variant’s spread across California and Mexico reported a peak in external introductions around late June 2021 (37). In comparison, the highest rate of introductions in Houston occurred approximately 20 d later. This lag in timing further supports our conclusion that Houston did not serve as a major entry point in the United States. Nevertheless, international introductions into Houston had a substantial impact, particularly during the early stages of the outbreak. These early events often led to larger and more persistent transmission clusters, posing significant challenges for public health response efforts.

Tip-trait association quantifies the degree to which viral phenotypic characters are correlated with shared ancestry, as represented by a viral phylogenetic tree (29). A common application of this phylogeny-trait correlation is to explore spatial structure (38); specifically, whether sequences group together in a phylogeny based on geographic location. In this study, we examine the correlations between phylogeny and population structures to better understand how demographic factors influence transmission dynamics. Human movements and interactions were generally more constrained by age group than by sex (39). For example, children are typically found in daycare centers or middle schools, teenagers in high schools, adults at their workplaces, and seniors in nursing homes. Our analysis on circulating clusters statistically supports that traits age groups are more tightly correlated with the tree topology, indicating more constrained transmission within these groups. Furthermore, we estimated the discrete trait model to describe the transmission between age groups. This analysis revealed that the primary sources of transmission to the vulnerable group—seniors—were teenagers, young adults, and middle-aged adults.

Previous reports showed the COVID-19 epidemic in Houston exhibited distinct patterns, including varying infection probabilities and hospitalization rates (22). We reconstructed the local dispersal history of SARS-CoV-2 across nine subregions of Greater Houston using discrete trait analysis. Because this approach can be influenced by sampling bias (40), we assessed the number of samples collected per county. A strong correlation between sample counts and both case and death counts suggests that sampling effort was roughly proportional to epidemic size.

We applied the Source Sink Score, Local Import Score, and Persistence Time to characterize transmission patterns in each subregion. Through Bayesian phylogeographic inference, we estimated the values of these metrics along with their HPD intervals, providing a measure of confidence in our estimates. Our analysis revealed a consistent pattern across all subregions: Regions with higher Source Sink Scores were associated with lower Local Import Scores and higher Persistent Times. This pattern aligns with previous analyses of viral transmission in Seattle (32), where a structured coalescent model (41) found that South King County exhibited longer persistence of local transmission compared to North King County, where external viral introductions drove a larger proportion of new cases.

Despite providing actionable public health information, our approach could be improved in several ways. We assume that the sequenced data from outside the focal area is representative of the global outbreak. The approach we used to detect transmission clusters depends on incorporating background sequences from other regions. Differences in sampling ratios may bias this detection. While we made efforts to address this, increased bias resulting from lower sampled epidemics or early phases of an outbreak may reduce sensitivity of this cluster identification. Simulation studies could help address this by evaluating how the sampling ratios of background and focal sequences influence cluster detection accuracy. Additionally, while we included genomic data alongside ZIP code, age, gender, and collection date, we lacked access to individual-level metadata on key transmission drivers such as household structure, contact rates, and occupational exposure. These social and behavioral factors likely influence cluster size and persistence but are difficult to assess without contact tracing or population survey data. Finally, we were unable to incorporate vaccination coverage into our analysis, despite its importance in shaping transmission dynamics and the role of specific counties as sources or sinks.

We determined whether a location functions as a source for regional spread. These findings collectively suggest that a well-established and sustained local epidemic is crucial for the onward spread of the pathogen to other areas. Focused strategies can enhance the efficiency of outbreak control measures. Public health interventions, such as temporary closures of schools, limitations on large public gatherings, enhanced testing and contact tracing, and increased access to healthcare resources, could influence local and regional transmission dynamics.

In summary, our study demonstrates the utility of integrating genomic epidemiology with spatial and demographic data to uncover fine-scale transmission patterns of SARS-CoV-2 within a large and diverse metropolitan area. By quantifying introduction events, identifying locally circulating clusters, and evaluating transmission heterogeneity across subregions and demographic groups, we provide insights that can inform more targeted and equitable public health interventions. As genomic surveillance continues to expand, combining it with detailed epidemiological and social data will be essential for improving outbreak response and preparedness for future public health threats.

## Materials and Methods

### SARS-CoV-2 Genomic Dataset.

With the support of the HHD, we accessed a large dataset of SARS-CoV-2 genomes sampled in Houston, along with linked metadata, including ZIP code, age group, and sex. The first reported Delta variant case in Houston occurred in mid-April 2021. Our contextual dataset (non-Houston) was divided into two phases: Phase one included all worldwide sequences available in GISAID (www.gisaid.org) sampled before April 15, while Phase two sampled 1% of worldwide sequences available after April 15 (*SI Appendix*, Fig. S7). This sampling scheme balanced the need to include early sequences with the practical limits of handling a rapidly growing dataset.

We began with a combined dataset of 32,734 Delta variant SARS-CoV-2 genomes from Houston and contextual sources. Sequences were aligned to the reference genome (GenBank ID: NC_045512.2) using minimap2 v2.24 (42). We excluded 5,356 low-quality sequences with mapped completeness below 93% and trimmed alignments outside the reference coordinates 265 to 29,674, padding the ends with Ns to mask the 5′ and 3′ UTRs. Next, we calculated the number of base-pair differences between each alignment and the reference genome. To preliminarily filter out sequences with poor molecular clock signal, we removed 1,240 outliers—defined as samples collected within the same Epi-Week that exhibited genetic distances greater than 3.0 SD from the weekly mean. This resulted in a final dataset of 26,138 high-quality alignments, including 9,186 from Houston, 5,334 from domestic (non-Houston United States) sources, and 11,618 from international sources (*SI Appendix*, Fig. S8).

### Time-Scaled Phylogenetic Inference.

Time-scaled phylogenies were inferred through the following steps: inferring an initial phylogenetic tree, calibrating it with time, and postprocessing to refine the final tree. First, a maximum-likelihood phylogeny was estimated using IQ-TREE v2.3.2 (43), applying the HKY85 nucleotide substitution model with empirical base frequencies. The phylogeny was rooted using early samples from Wuhan (Wuhan-Hu-1/2019) to reflect the epidemic’s origins. Next, time calibration was performed using TreeTime v0.11.2 (44), which estimated an evolutionary rate of 0.0007 substitutions per site per year with r^2 = 0.35. A total of 102 tips with residuals greater than three interquartile distances from the residual distribution were flagged as outliers. Finally, the tree underwent postprocessing steps. Outlier pruning was conducted using jclusterfunk v0.0.25 (https://github.com/rambaut/jclusterfunk). The tree format was subsequently standardized using gotree v0.4.5 (45) to ensure compatibility for downstream analyses.

### Introduction Analysis.

Before considering a discrete trait model to jointly reconstruct viral spread between locations and between age groups, we performed a preliminary discrete phylogeographic analysis (24) on the fixed time-scaled tree obtained in the previous step to identify independent introduction events. Tips of the tree were assigned locations of either Houston, “Domestic”, or “International”. After reconstructing the ancestral locations of the virus, we identified introduction events on branches connecting non-Houston nodes (categorized as either Domestic or International) to Houston nodes or tips (Fig. 1*C*). The introduction time was defined as the midpoint of these branches. Locally circulating clusters were then extracted by 1) extending pseudoclusters from introduction events to encompass all nodes and tips assigned to Houston and 2) refining these clusters by removing nodes with no descendants and collapsing branches connected to single-descent nodes (Fig. 1 *D*–*F*). This algorithm ensures that each isolate from Houston is assigned to a cluster. Phylogeny reading, editing, writing, and visualization were performed using the R packages ape v5.8 (46), treeio v1.20.2 (47), tidytree v0.4.6 (48), and ggtree v1.14.6 (49), as well as the Python library Baltic v0.3.0 (https://github.com/evogytis/baltic).

Based on the observed distribution of cluster sizes (assumed to follow a negative binomial distribution), we estimated the mean cluster size (r) and the dispersion parameter (k). Maximum likelihood estimation was performed using the “fitdistr” function from the MASS package in R (50).

Time-scaled phylogenies of locally circulating clusters with more than 50 sequences were used as input for Phylodeep v0.9 (27) to infer epidemiological parameters. The analysis assumed a sampling probability of 0.1, employed the birth-death model, and utilized feed-forward neural networks (FFNN).

We simulated 5 Markov chains of 20 million states each, with a burn-in of 4 million states, using BEAST v1.10.5 (51) with XML input files generated using custom R scripts. Trees were sampled every 160,000 states, yielding a posterior tree distribution of 500 trees. The convergence and mixing of all relevant parameters were inspected using Tracer v1.7 (52). We analyzed the posterior tree distribution to infer 95% HPD intervals for the weekly count of introduction events, the mean cluster size (r) and the dispersion parameter (k). A representative tree was selected from the posterior tree set, matching the posterior median for total introductions (*SI Appendix*, Fig. S9). From this tree, we extracted 82 locally circulating clusters, each comprising more than 10 isolates.

### Tip-Trait Association Test.

For each locally circulating cluster, we quantified phylogeny-trait associations using the Association Index (29, 30). The Association Index accounts for both the phylogenetic topology and the trait assignments at the tips, with lower values indicating stronger clustering of identical traits—reflecting a stronger association between the trait and the phylogeny. To assess the significance of these correlations, we generated null distributions of the Association Index by randomly permuting tip trait assignments 1,000 times. This enabled a tip-trait association test. All scripts for this test were bundled into an R package named TTAT, which is publicly available on GitHub at https://github.com/leke-lyu/TTAT.

### Jointly Fitted Discrete Trait Model.

We modeled the transition of discrete traits on local clusters using a continuous-time Markov chain, analogous to standard approaches for modeling molecular sequence evolution (24). Assuming that all 82 circulating clusters shared the same population process, we jointly estimated two separate transition rate matrices (36, 53, 54): one describing transitions between age groups and another describing transitions between counties. The age group model included five distinct categories: infants and children, teenagers, young adults, middle-aged adults, and seniors. We assumed a nonreversible transition model (55) consisting of 20 separate rate parameters, each augmented with a binary indicator variable to perform Bayesian stochastic search variable selection (BSSVS) (56). Similarly, the geographic model analyzed transitions between 9 counties, applying a nonreversible transition model with 72 separate rate parameters, each accompanied by a BSSVS indicator variable.

For both models, a Poisson prior with a mean of 1 and an offset of 0 was used for the total number of nonzero rates. XML configuration files for the BEAST runs were generated using custom R scripts. We performed five independent chains, each consisting of 100 million states with a burn-in period of 20 million states. Trees were sampled every 800,000 states, yielding a posterior tree distribution of 500 samples per cluster. Convergence and mixing were verified using Tracer.

The geographic model was further extended with a GLM (25), where viral diffusion rates among counties served as the outcome in a log-linear combination of epidemiological predictors, regression coefficients, and indicator variables for BSSVS. Population size, population density, and median household income for all nine counties were obtained from the US Census Bureau. Accumulated death count and case count were obtained from the Texas COVID-19 Surveillance Archives. To assess collinearity among predictors, we conducted PCA and calculated pairwise Pearson correlation coefficients, which informed the final predictor selection.

### Posterior Processing.

Using a jointly fitted discrete trait model, we reconstructed the geographic states of internal nodes, yielding posterior tree distributions for 82 locally circulating clusters. For each subregion in Houston, we treated it as the focal area and characterized phylogenetic branches associated with it as imports, local transmissions, or exports, based on the inferred geographic states of connected nodes. Specifically, imports were defined as branches connecting a node in another area to a node or tip in the focal area; local transmissions were defined as branches connecting two nodes within the focal area; and exports were defined as branches connecting a node in the focal area to a node or tip in another area. Using the entire outbreak period as the time window, the counts of imports, local transmissions, and exports were used to calculate the Source Sink Score and the Local Import Score (31), two epidemiological metrics that summarize the transmission patterns of each subregion.

The Source Sink Score was defined as the difference between the number of exports and imports, normalized by their sum. The score ranges from –1 to 1, with values closer to 1 indicating that the subregion predominantly acted as a viral source, and values closer to –1 indicating that it primarily functioned as a viral sink. The Local Import Score was defined as the number of imports divided by the sum of local transmissions and imports. This score ranges from 0 to 1, with higher values indicating that the outbreak in a subregion was primarily maintained by repeated introductions, and lower values suggesting a transition to sustained local transmission.

We also calculated the Persistence Time (32–34) to quantify how long a viral lineage circulated within a subregion. For each tip, we traced backward along the phylogeny to identify the first ancestral node inferred to be in a different location.

## Supplementary Material

Appendix 01 (PDF)

## Data Availability

GISAID accession IDs and demographic metadata data (sampling date, location, age, and sex) have been deposited in Dryad (57).
