# Supplementary material for: Tracing SARS-CoV-2 clusters across local scales using genomic data
Source: Proc Natl Acad Sci U S A. 2025 Aug 7;122(32):e2501435122. doi: 10.1073/pnas.2501435122 (PMC12358902; doi:10.1073/pnas.2501435122)
Supplement: Supplementary file 1 — Appendix 01 (PDF) [file pnas.2501435122.sapp.pdf]

PNAS

Supporting Information for
Tracing SARS-CoV-2 Clusters Across Local Scales Using Genomic Data

Leke Lyu[a], Mandev Gill[b], Guppy Stott[a], Sachin Subedi[a], Cody Dailey[a], Gabriella Veytsel[a], Magdy Alabady[c], Kayo Fujimoto[d], Ryker Penn[e], Pamela Brown[e], Roger Sealy[e], Justin Bahl[a]*

a. Center for Ecology of Infectious Diseases, Institute of Bioinformatics, Department of Infectious Diseases, Department of Epidemiology and Biostatistics, University of Georgia, Athens, GA, USA

b. Department of Statistics, Institute of Bioinformatics, Center for Ecology of Infectious Diseases, University of Georgia, Athens, GA, USA

c. Georgia Genomics and Bioinformatics Center, University of Georgia, Athens, GA, USA

d. Department of Health Promotion and Behavioral Sciences, The University of Texas Health Science Center at Houston, Houston, TX, USA

e. Houston Health Department, Houston, TX, USA

Corresponding Author: *Justin Bahl
Email: justin.bahl@uga.edu

This PDF file includes:

Figures S1 to S9
Tables S1 to S4

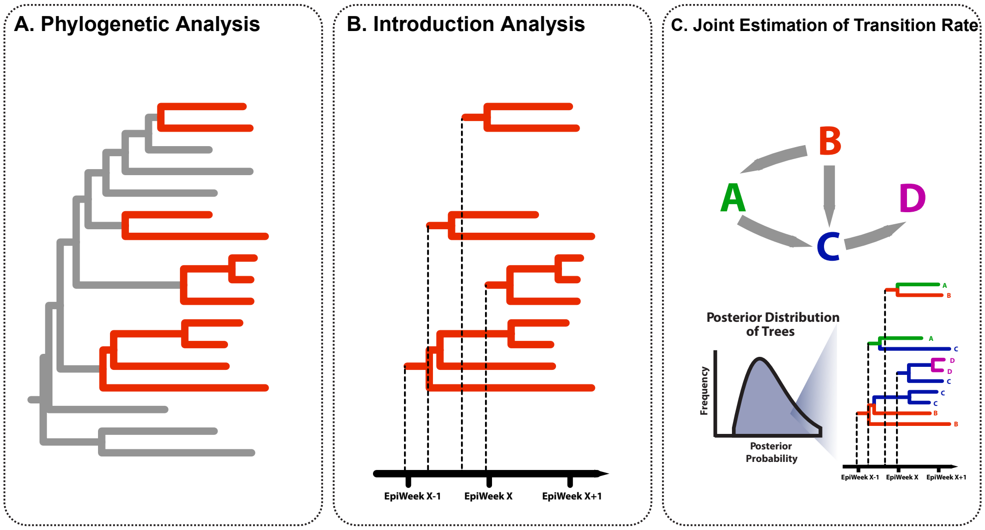

Fig. S1. Conceptual Workflow of Large-Scale Genomic Epidemiology Analysis.

Phylogenetic Analysis infers the phylogeny representing the evolutionary history of the isolated sampled from the focal region within a global context. Introduction analysis estimates the timing of viral introductions and identifies locally circulating clusters. The Joint Fit Model infers transition rates under a unified transition matrix.

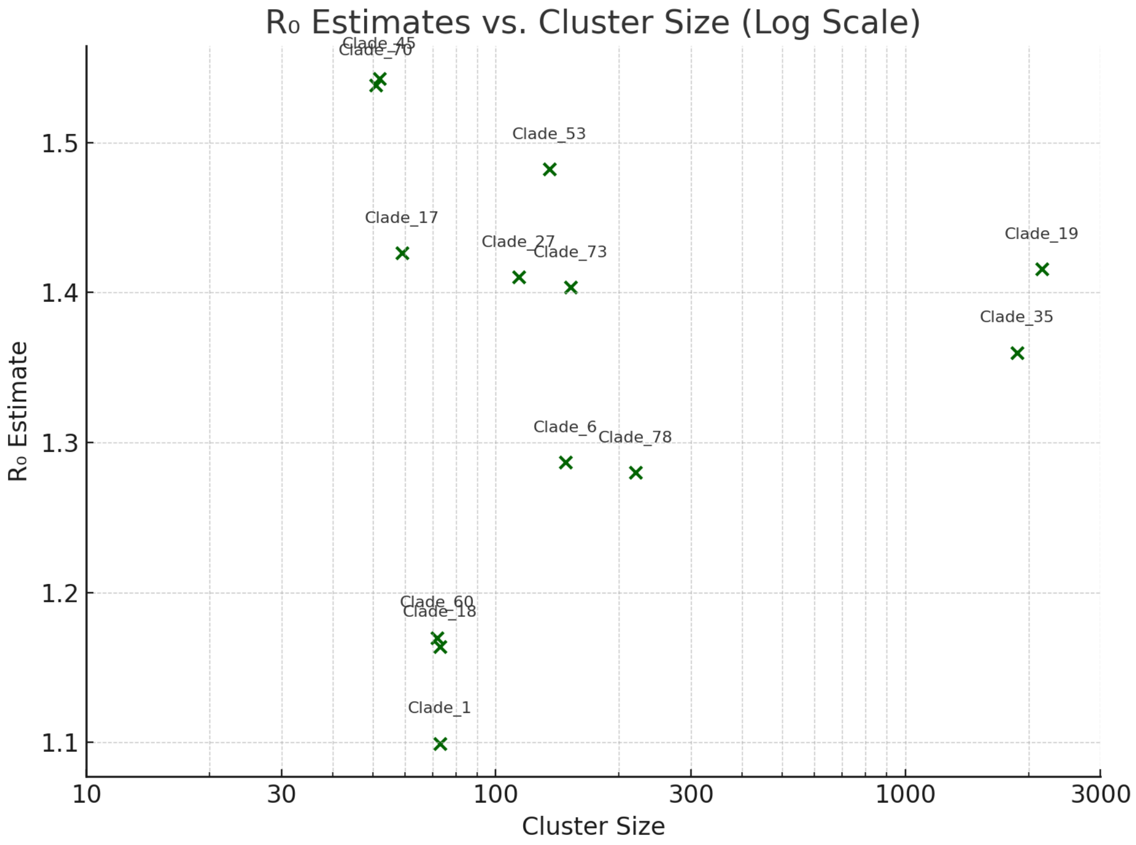

Fig. S2. Estimated basic reproduction numbers (R_0) for selected locally circulating clusters, inferred using Phylodeep.

The x-axis represents cluster size on a log scale, and the y-axis shows the estimated R_0 values. Each point corresponds to a distinct cluster. Notably, the two largest clusters do not exhibit the highest R_0 values, suggesting that larger cluster size does not necessarily reflect higher transmissibility.

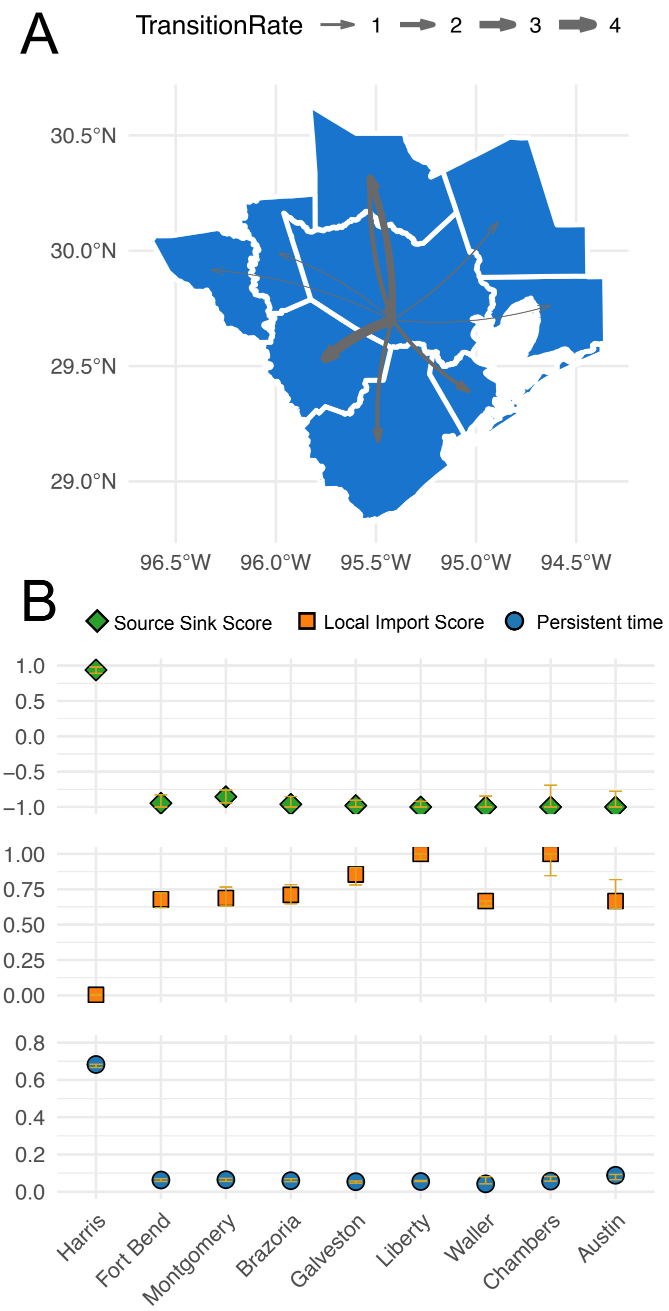

Figure S3. Distinct Transmission Patterns in Subregions of Greater Houston (Joint Estimates Based on the Two Largest Clusters).

A. Discrete phylogeographic reconstruction of the dispersal history across 9 counties. Arrow thickness indicates the magnitude of transition rates. All transitions shown on the map were decisively supported by Bayes factors (>100) **B.** Source Sink Scores, Local Import Scores, and Persistence Time across these subareas. In these bar charts, golden error bars represent the associated 95% HPD, providing a measure of uncertainty for each score. Source Sink Score ranges from 1 (viral source) to -1 (viral sink). Local Import Score ranges from 0 (epidemic is locally maintained) to 1 (epidemic relies on introduction).

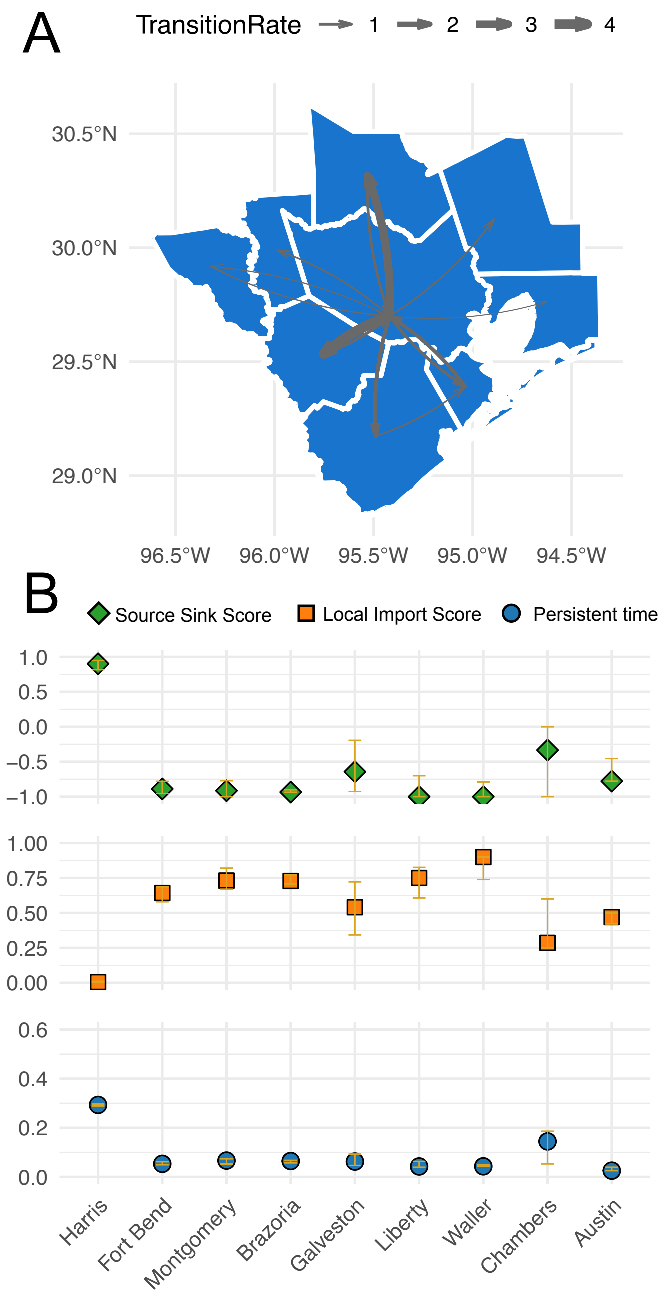

Figure S4. Distinct Transmission Patterns in Subregions of Greater Houston (Joint Estimates Based on the Remaining 80 Smaller Clusters).

A. Discrete phylogeographic reconstruction of the dispersal history across 9 counties. Arrow thickness indicates the magnitude of transition rates. All transitions shown on the map were decisively supported by Bayes factors (>100) **B.** Source Sink Scores, Local Import Scores, and Persistence Time across these subareas. In these bar charts, golden error bars represent the associated 95% HPD, providing a measure of uncertainty for each score. Source Sink Score ranges from 1 (viral source) to -1 (viral sink). Local Import Score ranges from 0 (epidemic is locally maintained) to 1 (epidemic relies on introduction).

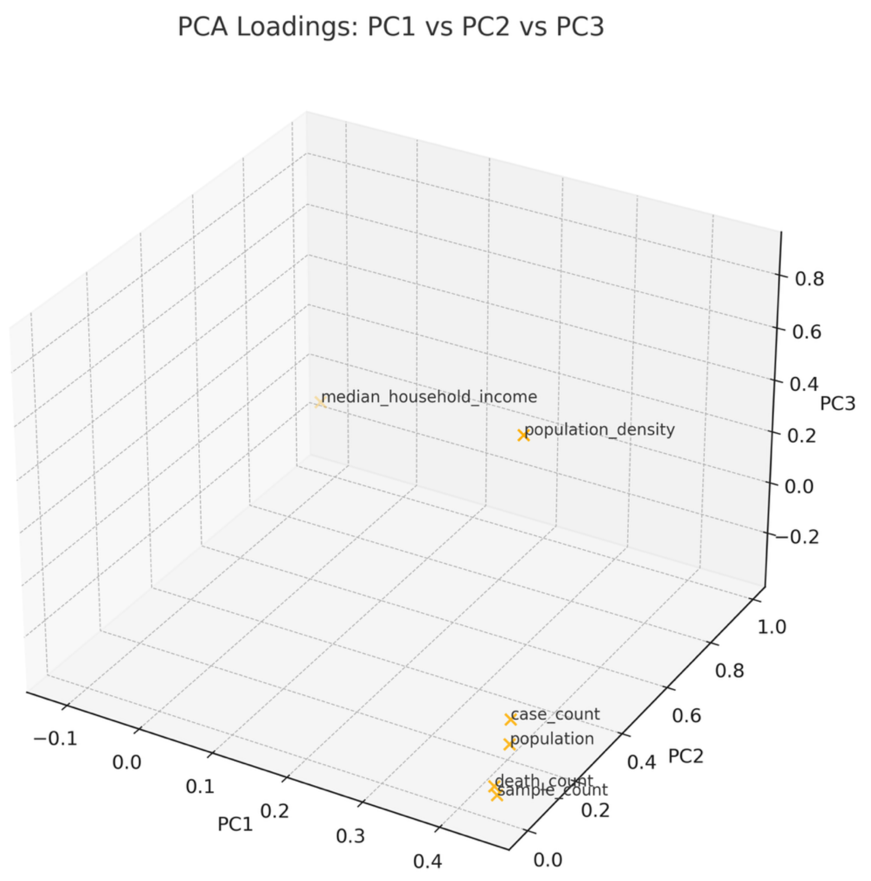

Figure S5. Principal component analysis (PCA) loadings for six county-level predictors used in the GLM.

Variables including sample count, case count, population size, and death count cluster closely together in the loading space, indicating strong collinearity.

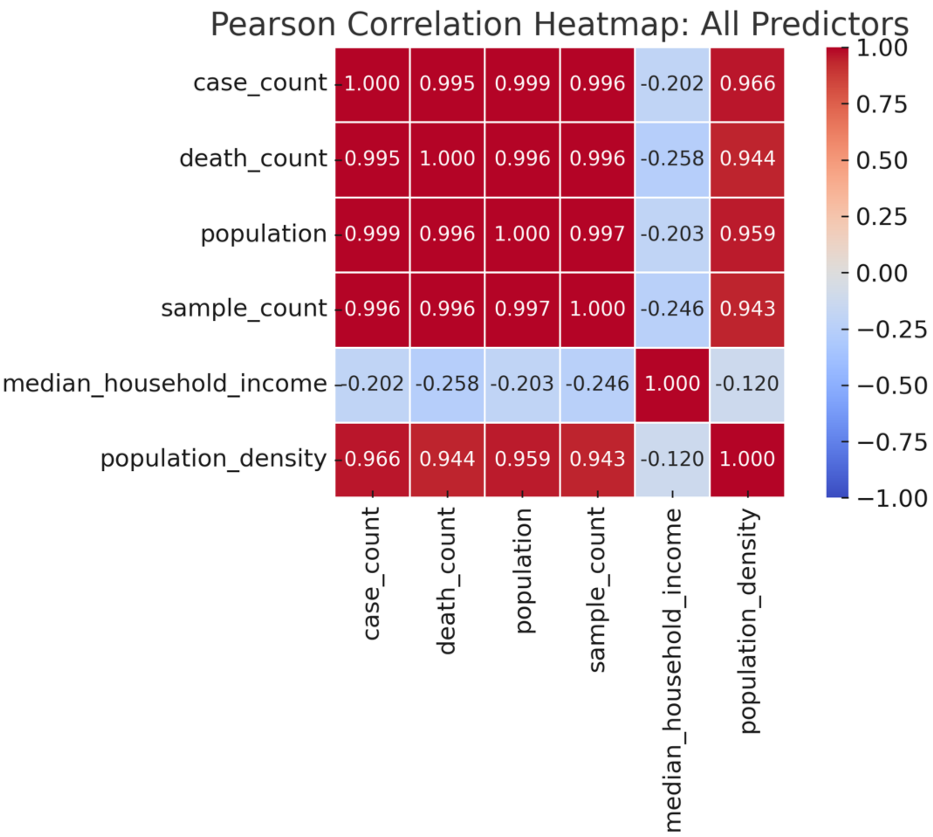

Figure S6. Pearson correlation heatmap of county-level predictors used in the GLM.

Red indicates strong positive correlation, while blue indicates strong negative correlation.

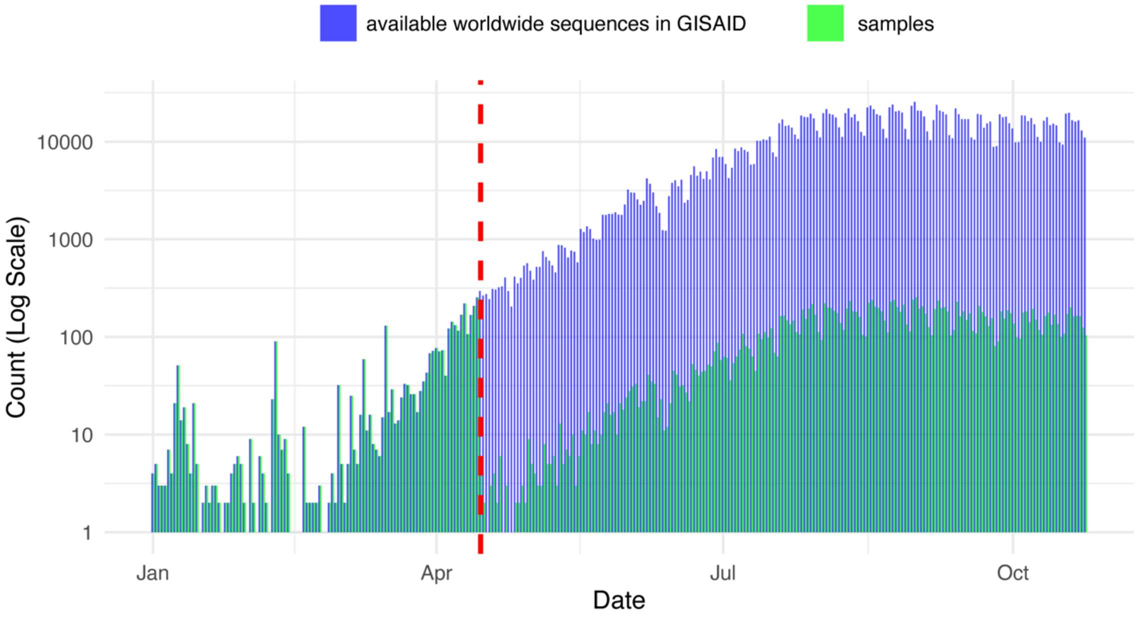

Fig. S7. Sampling scheme for contextual sequences.

The contextual sequence dataset includes all worldwide sequences available in GISAID (www.gisaid.org) sampled before April 15, as well as 1% of the worldwide sequences available after April 15.

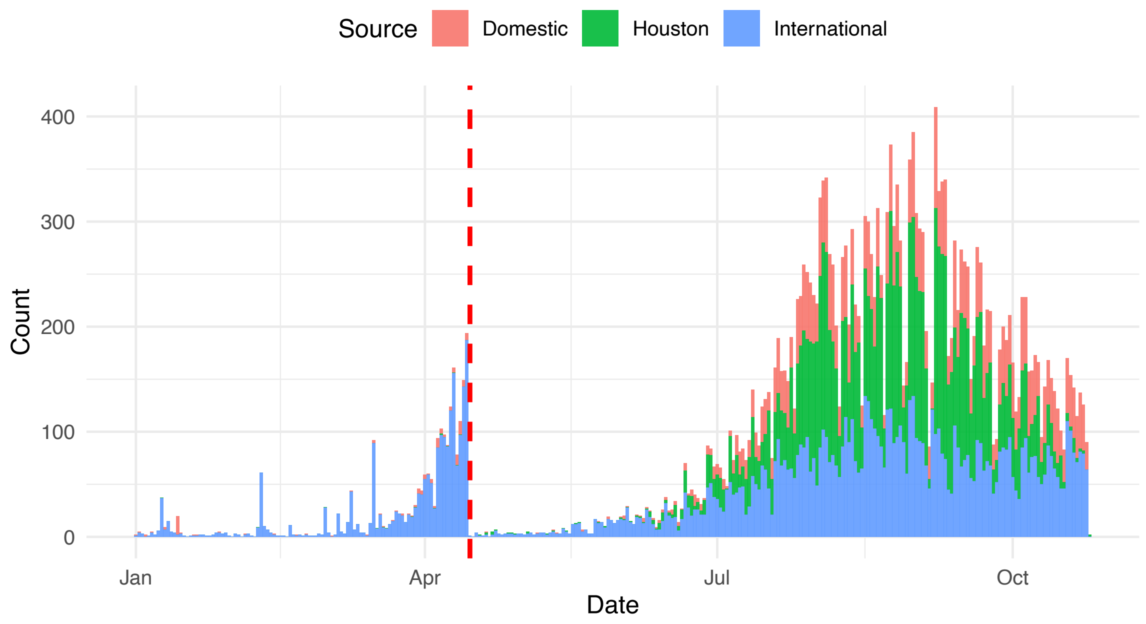

Fig. S8. Sampling date distribution of the complete genome dataset.

The bars in the plot represent the daily count of isolates, categorized by source (international, domestic, and Houston) and distinguished by color.

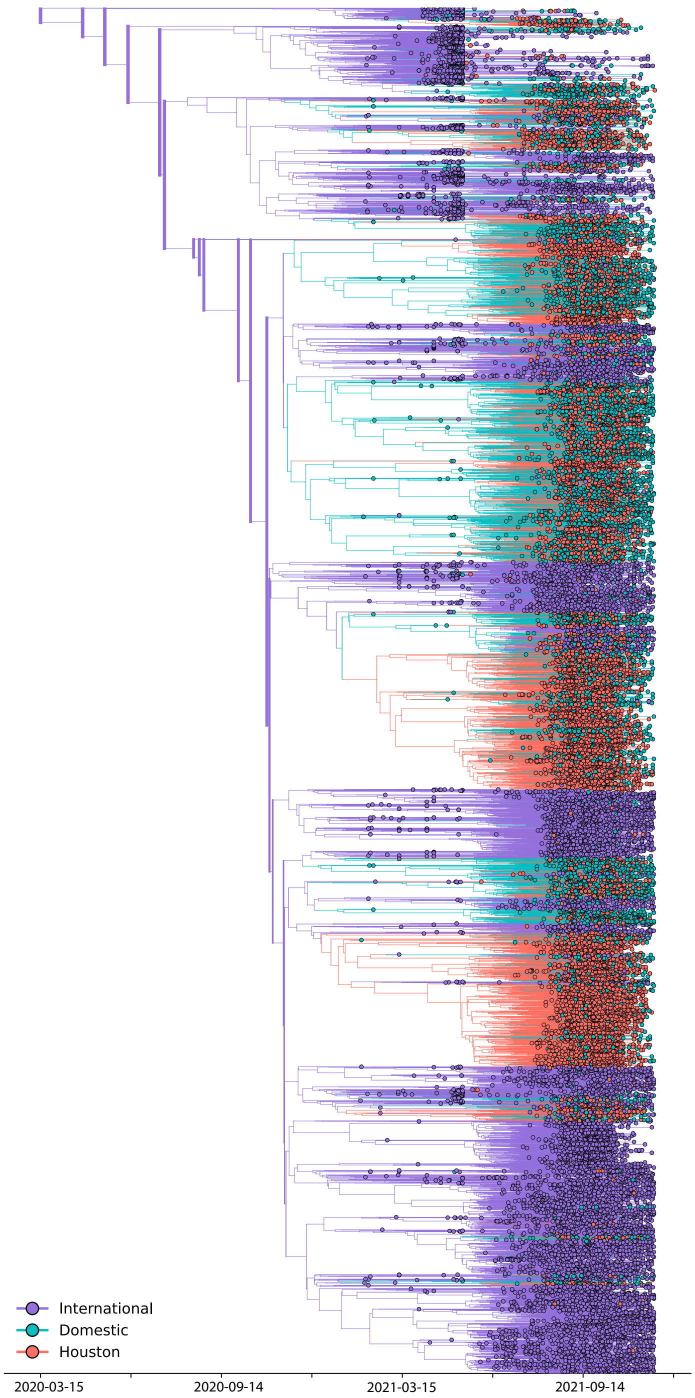

Fig. S9. Time-scaled phylogenetic tree of SARS-CoV-2 genomes sampled in Houston and beyond.

Tips represent individual genomes, colored by sampling location: purple (international), blue (domestic, non-Houston), and red (Houston). Internal branches are colored by inferred node location to highlight introductions and local transmission. Vertical branches are scaled by the number of descendant tips to reflect cluster size. The x-axis represents time.

Table S1. Estimated Epidemiological Parameters for Selected Locally Circulating Clusters, Inferred Using Phylodeep.

Cluster ID	Size	R_0	Infectious Period (year)
Cluster_1	73	1.099	0.037
Cluster_17	59	1.426	0.078
Cluster_18	73	1.164	0.051
Cluster_19	2159	1.416	0.069
Cluster_27	114	1.41	0.083
Cluster_35	1878	1.36	0.069
Cluster_45	52	1.542	0.071
Cluster_53	135	1.482	0.074
Cluster_6	148	1.287	0.068
Cluster_60	72	1.17	0.044
Cluster_70	51	1.538	0.061
Cluster_73	152	1.403	0.069
Cluster_78	219	1.28	0.06

Table S2. Transmission rates between age groups.

Age groups were categorized as follows: infants and children (0–12 years), teenagers (13–18 years), young adults (19–35 years), middle-aged adults (36–55 years), and seniors (56 years and older). All transmissions shown here have an inclusion probability > 0.5. The Bayes factors are provided for each rate.

From	To	Transition Rate	BF
Young Adults	Middle-aged Adults	4.813	> 10,000
Middle-aged Adults	Young Adults	2.216	> 10,000
Middle-aged Adults	Infants and Children	1.895	> 10,000
Middle-aged Adults	Seniors	1.518	> 10,000
Middle-aged Adults	Teenagers	1.516	> 10,000
Young Adults	Seniors	1.459	> 10,000
Young Adults	Teenagers	1.216	> 10,000
Young Adults	Infants and Children	1.202	> 10,000
Teenagers	Middle-aged Adults	0.314	3258.597
Seniors	Middle-aged Adults	0.234	51.837
Teenagers	Seniors	0.229	> 10,000
Infants and Children	Young Adults	0.214	> 10,000
Seniors	Infants and Children	0.208	72.596
Seniors	Teenagers	0.193	1915.479
Infants and Children	Teenagers	0.180	1479.402
Teenagers	Infants and Children	0.163	122.195
Infants and Children	Middle-aged Adults	0.156	13.449
Seniors	Young Adults	0.138	5.430

Table S3. Transmission rates between counties.

All transmissions shown here have an inclusion probability > 0.5. The Bayes factors are provided for each rate. A dash ("–") indicates that the corresponding transition did not meet the inclusion probability threshold (>0.5).

From	To	All 82 Clusters		2 Largest Clusters		The Rest 80 Clusters	
		Transition Rate	BF	Transition Rate	BF	Transition Rate	BF
Harris	Fort Bend	4.569	> 10,000	4.209	> 10,000	4.186	> 10,000
Harris	Montgomery	3.292	> 10,000	2.843	> 10,000	3.382	> 10,000
Harris	Brazoria	1.642	> 10,000	1.510	> 10,000	1.482	> 10,000
Montgomery	Harris	1.455	> 10,000	1.812	> 10,000	0.957	801.872
Harris	Galveston	1.373	> 10,000	1.445	> 10,000	1.070	> 10,000
Fort Bend	Harris	1.147	> 10,000	1.044	43.290	1.073	> 10,000
Galveston	Harris	0.546	> 10,000	-	-	1.344	> 10,000
Fort Bend	Galveston	0.460	166.107	-	-	0.382	20.727
Harris	Liberty	0.423	> 10,000	0.355	> 10,000	0.464	> 10,000
Harris	Waller	0.301	> 10,000	0.189	> 10,000	0.430	> 10,000
Brazoria	Galveston	0.214	> 10,000	-	-	0.393	> 10,000
Harris	Chambers	0.170	> 10,000	0.171	> 10,000	0.141	323.735
Harris	Austin	0.169	> 10,000	0.132	> 10,000	0.220	> 10,000
Chambers	Harris	0.166	9.614	-	-	0.344	26.123
Austin	Harris	0.139	7275.103	-	-	0.266	7275.104

Table S4. Predictors used in the GLM

County	Case	Population	Population Density	Median Household Income	Death	Sample
Austin	2,140	30,167	46.7	68,630	43	22
Brazoria	36,372	372,031	274.0	86,197	625	192
Chambers	4,606	46,571	78.0	102,146	48	19
Fort Bend	72,153	822,779	955.1	105,583	603	568
Galveston	40,631	350,682	926.8	75,947	505	169
Harris	398,729	4,731,145	2777.3	68,748	6152	5019
Liberty	7,280	91,628	79.1	61,880	265	45
Montgomery	50,725	620,443	595.6	95,241	855	387
Waller	3,886	56,794	110.6	75,223	66	34
